# Endovascular Management of Middle Aortic Syndrome Presenting with Uncontrolled Hypertension

**DOI:** 10.1155/2018/9586025

**Published:** 2018-10-28

**Authors:** Owen S. Glotzer, Kathryn Bowser, F. Todd Harad, Sandra Weiss

**Affiliations:** The Heart and Vascular Center, Christiana Care, Newark, DE 19713, USA

## Abstract

Middle Aortic Syndrome is a rare vascular disorder consisting of narrowing or stenosis of the distal thoracic or abdominal aorta. It is described in the literature in the form of case studies and case series. The authors present an unusual case of Middle Aortic Syndrome attributed to Takayasu's arteritis in a 60-year-old female who presented to the emergency department with uncontrolled hypertension. Traditional intervention involves open surgical bypass. This case study reviews the published literature on this rare syndrome and illustrates a successful alternative to open surgery through an endovascular approach.

## 1. Introduction

Middle Aortic Syndrome is a rare vascular disorder consisting of narrowing or stenosis of the distal thoracic or abdominal aorta. This constellation of anatomic abnormalities and symptoms is generally attributed to either a developmental/congenital disorder or to inflammatory vasculitis such as Takayasu's arteritis (TAK). Organ and muscle malperfusion distal to the affected aorta can lead most commonly to lower extremity claudication, resistant hypertension, and abdominal pain [1, 2]. It is classically identified during the first three decades of life and has been traditionally treated with open surgery [3–6]. The authors present a unique case of severe abdominal aortic stenosis in a 60-year-old female who presented with uncontrolled hypertension.

## 2. Case Report

A 60-year-old female presented to the outpatient cardiology clinic for evaluation of worsening chronic hypertension for which she had been on hydrochlorothiazide/Valsartan for 10 years. Her systolic blood pressure exceeded 200 mmHg in the office, and aggressive medical therapy was initiated in the outpatient setting. She returned to the emergency department the following day with headache and malaise and systolic blood pressure above 200 mmHg for which she was treated and discharged.

She returned to the emergency department again 5 days later, this time with complaints of word finding difficulty, blurred vision, and lower extremity tingling. Her blood pressure on presentation was 216/81 mmHg. She was admitted, and workup demonstrated no acute intracranial process or carotid stenosis. Echocardiography revealed mild concentric left ventricular hypertrophy with a preserved ejection fraction. Her blood pressure continued to be refractory to medical therapy despite five antihypertensive agents and eventual initiation of an esmolol infusion. A renal artery ultrasound identified renal artery stenosis with flow at the arterial origin measuring 350 cm/s on the right and 208 cm/s on the left (Table 1); flow velocity in the supraceliac aorta was also noted to be elevated. She had no history of kidney disease and no elevation of her creatinine. Vascular surgery was consulted and a history of lower extremity claudication was elicited. On exam she had weak but palpable femoral pulses and an audible abdominal aortic bruit; ankle-brachial index measurements were deferred and the patient was scheduled for angiogram.

**Figure 1:**
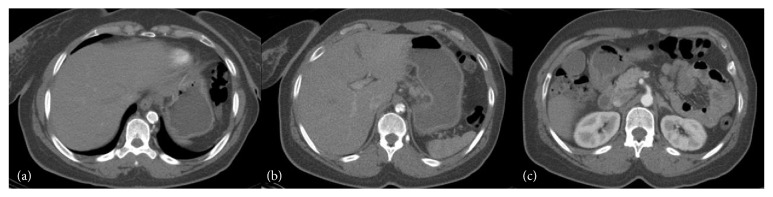
(a) Aorta proximal to stenosis with circumferential calcification measuring 9 mm lumen and 14 mm external diameter. (b) Aorta at the level stenosis with a luminal diameter of 5 mm. (c) Aorta distal to stenosis with a 14 mm wall and a patent lumen.

**Figure 2:**
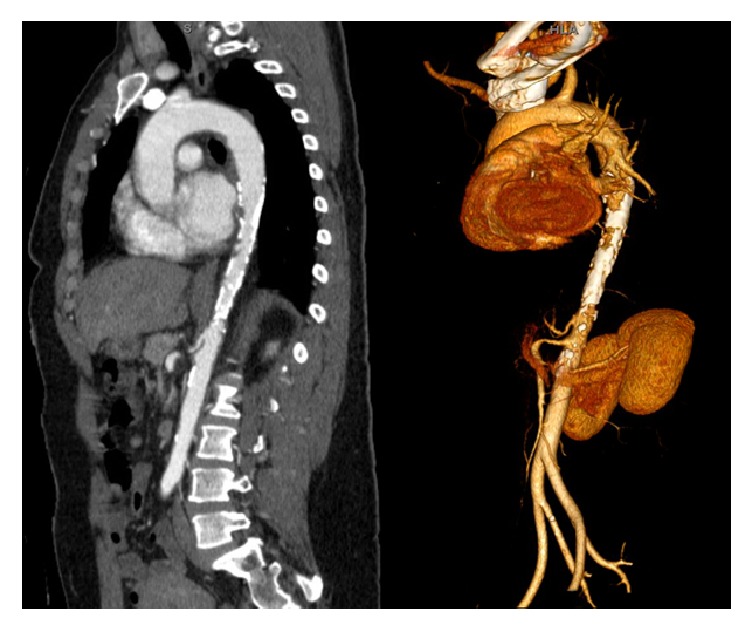
CTA of showing the area of stenosis in the supraceliac aorta with 3D reconstruction.

The patient underwent aortography the following day and on selective angiography the renal arteries were found to be widely patent. Significant stenosis was identified at the distal thoracic aorta extending into the abdominal aorta but terminating proximal to the celiac trunk. The degree of stenosis was deemed to be greater than 90% and a pressure gradient between the upper extremity and intra-aortic measurements exceeded 100 mmHg. CTA was subsequently performed to evaluate the extent of the lesion and confirmed a stenosis 1.3 cm proximal to the celiac origin measuring 5 mm at its narrowest point (Figures 1 and 2). The patient underwent arteriogram; the stenosis was successfully navigated and a Protege 14 × 40 x 12 mm nitinol stent (Medtronic Vascular, Santa Rosa CA) was delivered followed by a 10 mm postdilation balloon. A completion arteriogram demonstrated excellent flow across the stent.

After stenting, the patients' systolic blood pressure was 140-160 mmHg, and she experienced resolution of her lower extremity claudication. She was discharged from the hospital on Aspirin and Plavix and a blood pressure regimen consisting of lisinopril, hydralazine, amlodipine, and carvedilol.

She was lost to follow-up until two years later when she returned to the hospital with a blood pressure of 220/85 mmHg, with complaints of chest discomfort. CTA demonstrated stenosis in the distal portion of the aortic stent. An angiogram was performed, and the stent was ballooned to 12 mm. Pressure gradient measurements taken before and after dilatation decreased from 60 mmHg to 20 mmHg. On follow-up one year later, she continued to experience excellent blood pressure control.

## 3. Discussion

Middle Aortic Syndrome is a rare clinical presentation with only case studies and case series reported in the literature [4, 5, 7–9]. There is no characteristic presentation, but this syndrome can lead to severe hypertension, diminished or absent femoral pulses, lower extremity claudication, incongruent extremity pressure measurements, and audible arterial bruits. Laboratory analysis can show elevation of inflammatory markers such as erythrocyte sedimentation rate (ESR) and C-reactive protein (CRP). [10]

The classic imaging findings of Middle Aortic Syndrome have been described as an hour glass shaped aorta identified on CT scan or arteriography. Classification of this syndrome is based on the most proximal portion of the aorta affected [5, 6]. Distal lesions are more often associated with renal and/or splanchnic arterial disease, which is consistent with the case presented here as the infraceliac aorta was of normal caliber and there was no associated narrowing in the renal or splanchnic vasculature.

It is difficult to attribute the location and degree of narrowing to atherosclerotic disease alone given her lack of risk factors such as obesity, diabetes mellitus, or smoking history and lack of arterial calcification in other areas. Attributing her stenosis to congenital anomaly seems even more implausible given her age at presentation. Although large vessel arteritis is generally diagnosed in patients before the age of 30, adult onset TAK is well described [3, 11–13]. According to the American College of Rheumatology, three of six criteria are required for the diagnosis of TAK: onset at or before 40 years of age, 10 mmHg difference in brachial pressures, decreased pulsation in a brachial artery, subclavian or aortic bruit, narrowing/occlusions of the aorta, its primary branches or major proximal arteries in the extremities, and claudication of the extremities [14–16]. Although ESR and CRP were never obtained during her workup, according to these criteria the patient meets criteria for TAK.

Another point of interest is the ultrasound demonstrating flow acceleration in the renal arteries, with a lack of renal stenosis present on arteriography or CT. The authors attribute this finding to poststenotic flow acceleration centered at the level of the renal arteries, which is further supported by follow-up duplex study that demonstrates decreased flow velocity through the renal arteries after placement of the aortic stent. The native aorta distal to the stenosed segment measured 14 mm; as such, increased velocity is expected through a 10 mm stent. Placement of a larger stent was thought to pose too high a risk for iatrogenic trauma.

Traditional intervention consists of open surgery with bypass grafting, interposition grafting, or patch aortoplasty [5, 11, 17–20]. However, given the extent and complexity of these procedures, mortality rates have been quoted as high as 13% [21]. More recently, endovascular intervention has been performed with positive results [2, 22–25]. The extent of the lesion, involvement of visceral and/or renal arteries, age, and comorbidities of the patient need to be taken into consideration when selecting the most appropriate therapy [26]. The patient described here achieved blood pressure control and experienced resolution of her associated symptoms; despite the need for reintervention the authors maintain that endovascular therapy was the appropriate approach in this instance. The longevity of aortic stenting for stenotic lesions has yet to be determined, and some patients will require reintervention, as was true in this case. The decreased risk of morbidity and mortality justifies the endovascular approach as the initial intervention in elderly patients or those with significant comorbidities.

## 4. Conclusion

Middle Aortic Syndrome is a rare entity with an often unclear clinical presentation. The authors of this manuscript add this presentation to the literature regarding hypertension stemming from significant stenosis of the abdominal aorta. This case study demonstrates that the endovascular approach should be viewed as an appropriate early step in the management of hypertension due to aortic stenosis for patients with anatomically amenable lesions, who are at an increased risk of complications from open surgery.

## Figures and Tables

**Table 1:** Renal artery duplex measurements.

Criteria	Right Kidney	Left Kidney
Size (cm)	8.71	10.26
Velocity (cm/s)		
Origin	350	208
Proximal	175	211
Mid	49	112
Distal	41	NR
RI		
Upper	0.64	0.59
Mid	0.69	0.57
Lower	0.71	0.50
RAR	2.69	1.67

*RI = resistive indices, RAR = renal to aortic ratio, and NR = not recorded*
